# Cryptocaryone Exhibits ROS/MAPK‐Dependent Antiproliferative and Apoptosis‐Inducing Effects on Triple‐Negative Breast Cancer Cells and Proof‐of‐Concept Breast Cancer Mouse Model

**DOI:** 10.1002/ddr.70286

**Published:** 2026-04-19

**Authors:** Ya‐Ting Chuang, Wangta Liu, Tsu‐Ming Chien, Ammad Ahmad Farooqi, Hsun‐Shuo Chang, Jun‐Ping Shiau, Hsueh‐Wei Chang

**Affiliations:** ^1^ Department of Biomedical Science and Environmental Biology PhD Program in Life Sciences, College of Life Science Kaohsiung Medical University Kaohsiung Taiwan; ^2^ Department of Biotechnology Kaohsiung Medical University Kaohsiung Taiwan; ^3^ School of Medicine Kaohsiung Medical University Kaohsiung Taiwan; ^4^ Department of Urology Kaohsiung Medical University Hospital Kaohsiung Taiwan; ^5^ Department of Urology Kaohsiung Gangshan Hospital Kaohsiung Medical University Kaohsiung Taiwan; ^6^ Department of Molecular Oncology Institute of Biomedical and Genetic Engineering (IBGE) Islamabad Pakistan; ^7^ School of Pharmacy, College of Pharmacy Kaohsiung Medical University Kaohsiung Taiwan; ^8^ Department of Surgery Division of Breast Oncology and Surgery, Kaohsiung Medical University Hospital Kaohsiung Medical University Kaohsiung Taiwan; ^9^ Center for Cancer Research, and Research Center for Molecular Medicine Kaohsiung Medical University Kaohsiung Taiwan; ^10^ Department of Medical Research Kaohsiung Medical University Hospital Kaohsiung Taiwan; ^11^ Drug Development and Value Creation Research Center Kaohsiung Medical University Kaohsiung Taiwan

**Keywords:** apoptosis, cryptocaryone, DNA damage, MAPK, triple‐negative breast cancer

## Abstract

Omics’ technologies have enabled clinicians to gain previously unprecedented insights into the molecular complexity and clinical heterogeneity of triple‐negative breast cancer (TNBC). Increasingly it is being realized that TNBC does not respond well to current targeted therapies. This study aims to explore the antiproliferative effects and cancer regulatory mechanisms which underlie the drug resistance and aggressiveness of TNBC cells. Cryptocaryone (CPC) derived from *Cryptocarya concinna* demonstrated antiproliferative responses to TNBC cells (HCC1937 and MDA‐MB‐231), while normal breast cells (H184B5F5/M10) exhibited low cytotoxicity. In an in vivo assessment, CPC effectively reduced tumor growth in the MDA‐MB‐231 xenografted mouse model without significantly affecting body weight. Mechanistically, CPC triggered apoptosis, as indicated by an increase in sub‐G1 and annexin V, as well as activated caspase 3 and 8. CPC also induced substantial oxidative stress by generating reactive oxygen species, mitochondrial superoxide, and membrane depolarization. CPC also induced oxidative DNA damage, as evidenced by the presence of γH2AX and 8‐hydroxy‐2‐deoxyguanosine, in TNBC cells. All these CPC‐induced changes were more pronounced in TNBC cells than normal cells. JNK and p38 MAPK inhibitors attenuate CPC‐induced antiproliferation in TNBC cells. CPC upregulates phosphorylated JNK and p38 in TNBC cells. *N*‐acetylcysteine pretreatment confirmed that oxidative stress plays a vital role in enhancing the antiproliferation, apoptosis, and DNA damage in TNBC cells. Moreover, the CPC‐upregulated apoptosis and caspase 3/8 activations in TNBC cells were inhibited by JNK and p38 inhibitors. The impact of ERK activation on antiproliferation and apoptosis was evident in MDA‐MB‐231 cells, but not in HCC1937 cells. In conclusion, CPC demonstrated antiproliferative effects on TNBC cells through apoptosis and DNA damage induced by oxidative stress and MAPK activation, while showing drug safety in normal cells and breast cancer mouse model.

## 1 Introduction

Breast cancer has the highest incidence among female cancers worldwide (Siegel et al. 2019). Most breast cancers show three main receptor types: estrogen receptor (ER), progesterone receptor (PR), and human epidermal growth factor receptor 2 (HER2). However, these three biomarkers are absent in a unique subset of breast cancers, called triple‐negative breast cancers (TNBCs) (Ismail‐Khan and Bui 2010), which account for 15%–20% of breast cancer cases (Jamdade et al. 2015).

Natural products provide potential anticancer drug resources for treating breast cancer (Androutsopoulos et al. 2008; Jin et al. 2024; Ke et al. 2022; Nagalingam et al. 2012). Some studies have focused on the antiproliferative effects of natural products on TNBC cells (Asadi‐Samani et al. 2019). For example, *Euphorbia szovitsii* extract had antiproliferation effects on TNBC cells (MDA‐MB‐231) (Asadi‐Samani et al. 2019). Screening for more natural products to treat TNBC cells is thus warranted.

*Cryptocarya* is a genus of evergreen trees (Family: Lauraceae). Several antiproliferative effects have been reported in oral, lung, colon, liver, lymphoma (Huang et al. 2014; Juliawaty et al. 2020; Wu et al. 2012; Xiong et al. 2021; Yang et al. 2017), and breast cancer cells (Fan et al. 2019; Widiyastuti et al. 2018) treated with extracts or pure compounds isolated from *Cryptocarya* plants. Cryptocaryone (CPC), the main bioactive component in *Cryptocarya* plants, has been identified in *C. konishii* (Kurniadewi et al. 2010), *C. rubra* (Ren et al. 2014), and *C. concinna* (Huang et al. 2014). Although the anticancer effects of CPC have been reported in leukemia (Kurniadewi et al. 2010) and colon (Ren et al. 2014) cancer cells, only cytotoxicity values (IC_50_) were provided without investigation of the anticancer mechanisms of CPC.

Oxidative stress modulations were a potential strategy for breast cancer treatment (Dong et al. 2025). For example, some natural products, such as aminoflavone (McLean et al. 2008), stenophyllol B (Lee et al. 2023), and excavatolide C (Shiau et al. 2025), induce oxidative stress to trigger apoptosis in breast cancer cells. Although CPC has been reported to trigger apoptosis in prostate (Chen et al. 2010) and liver (Yu et al. 2023) cancer cells, the role of oxidative stress in its anticancer effects was not investigated. Notably, the oxidative stress and antiproliferative effects of CPC on breast cancer cells have not been reported, especially not in TNBC cells.

Moreover, mitogen‐activated protein kinases (MAPKs) are modulated by oxidative stress (Jeong et al. 2014; Runchel et al. 2011). However, the contribution of the MAPKs to the modulation of antiproliferative effects by CPC has not been explored. Notably, CPC has been observed to have antiproliferation effects on some cancer cells without examining its in vivo function (Chang et al. 2016; Chen et al. 2010; Ren et al. 2014; Yu et al. 2023).

The present study examined the in vitro and in vivo antiproliferative effects of CPC on TNBC cells. It investigated the anticancer mechanisms of cellular and mitochondrial oxidative stress, apoptosis signaling, oxidative DNA damage, and MAPK signaling in cell models.

## 2 Materials and Methods

### 2.1 CPC Preparation and Reagents

The isolation of *C. concinna* root‐derived CPC (MW: 282.29 g/mol; CAS. 39012‐05‐0) was described in our previous work (Chang et al. 2016). The chemical structure and purity of CPC were confirmed via NMR. The mechanisms involving oxidative stress and apoptosis in CPC‐treated oral cancer cells were examined via their inhibitors, such as *N*‐acetylcysteine (NAC; 10 mM, 1 h pretreatment) (Sigma‐Aldrich, St. Louis, MO, USA) (Huang et al. 2018; Wang et al. 2018) and Z‐VAD‐FMK (ZVAD; 50 μM, 2 h pretreatment) (Selleckchem. com; Houston, TX, USA) (Chang et al. 2015). Inhibitors of mitogen‐activated protein kinases (MAPKs), such as c‐Jun N‐terminal kinase (JNK) (JNKi; SP600125, #M2076, 5 μM), p38 (p38i; SB203580, #M1781, 10 μM), and extracellular signal‐regulated kinase 1/2 (ERK1/2) (ERKi; PD98059, #M1822, 20 μM) (Abmole BioScience, Houston, Texas), were pretreated for 1 h before CPC treatment.

### 2.2 Cell Culture and Viability Assay

ATCC human TNBC cells (MDA‐MB‐231 and HCC1937) were included and maintained in mixed Dulbecco's Modified Eagle's Medium (DMEM)/F12 medium (3:2) and RPMI Medium 1640 (Gibco). Normal cells, H184B5F5/M10 (M10) (Jen et al. 2024) (Bioresource Collection and Research Center (BCRC), were maintained in Alpha medium. In addition to the medium, all cell lines were supplemented with 10% fetal bovine serum and antibiotics (50 U/mL penicillin and 50 μg/mL streptomycin). According to the manufacturer's instructions, an ATP assay (#6016941, PerkinElmer Life Sciences, Boston, MA, USA) was conducted to measure cell viability (Yang et al. 2015).

### 2.3 Animal Study

Six‐ to eight‐week‐old NOD/SCID female mice weighing 18–28 g were used. A total of eighteen mice were obtained from BioLASCO Taiwan Co., LTD. The animal experiments were approved by the Institutional Animal Care and Use Committee (IACUC) of Kaohsiung Medical University (No. 113055) and complied with the institutional guidelines. A xenograft mouse model was established via the subcutaneous inoculation of tumor cells. The mice were randomized into three groups: a vehicle group (*n* = 6), 200 μg CPC group (*n* = 6), and 50 μg CPC group (*n* = 6). Next, 10^7^ MDA‐MB‐231 cells were suspended in PBS with 0, 50, and 200 μg CPC, achieving a total volume of 100 μL, and then mixed with Matrigel (Chen et al. 2022; Fridman et al. 1990; Mullen et al. 1996; White et al. 2001; Yao et al. 2024) (ratio 1:1) for transplantation (final volume 0.2 mL) into the right flank. Body weight and tumor diameter were measured twice per week. Tumor volume was calculated using the ellipsoid equation according to the records ((length × width × width)/2). The mice were sacrificed at the end of this study (5 weeks), and tumor weights were recorded.

### 2.4 Cell Cycle Analysis

The cell cycle phase was determined by cellular DNA content staining with 7‐aminoactinmycin D (7AAD; 1 μg/mL, 30 min) [#40037] (Biotium) (Shiau et al. 2022). Stained cells were analyzed using flow cytometry (Guava easyCyte).

### 2.5 Double‐Staining Apoptosis Assays

Double staining with an annexin V/7AAD kit [#AVK050] (Strong Biotech) was applied to the CPC‐treated cells for apoptosis detection (Shiau et al. 2022). Annexin V‐positive populations, i.e., annexin V (+)/7ADD (+/−), were detected using a flow cytometry analysis and indicated apoptotic cells.

### 2.6 Western Blotting, Flow Cytometry, and Luminescence Assays for Apoptotic Signaling

40 μg protein extracts per treatment were used for Western blotting. The expressions of apoptotic signaling proteins, such as poly(ADP‐ribose) polymerase (PARP) and caspases 3, 8, and 9, were detected using Western blotting analysis (13.2% SDS‐PAGE and PVDF membrane transfer), as described in (Chien et al. 2024). Antibodies (1:1000) were derived from an apoptosis sampler antibody kit [#9915] and a cleaved caspase 8 [#9496] (Cell Signaling Technology), accompanied by the internal control antibody GAPDH (1:10000) [#GT239] (GeneTex). The band intensity was analyzed using ImageJ.

Moreover, peptide‐based caspase 3 and 8 activation kits [#A304R1G‐5 and #CPL8R1E‐5] (OncoImmunin) were used to quantify their activation via flow cytometry as described (Chuang et al. 2025). For luminescence assays, caspase‐Glo 3/7 and caspase‐Glo 8 luminescence kits (#G8090 and #G8201) (Promega) were used to detect their activations as described (Chuang et al. 2025) and analyzed by a luminometer (Berthold Technologies GmbH).

### 2.7 Reactive Oxygen Species (ROS) and Mitochondrial Superoxide (MitoSOX) Assay

2′,7′‐Dichlorodihydrofluorescein diacetate (DCFH‐DA; 2 μM, 30 min) [#D6883‐50MG] (Sigma‐Aldrich), a ROS‐sensitive probe, was used for ROS detection (Shiau et al. 2022; Shih et al. 2022). A high level of DCFH‐DA‐stained cells was considered indicative of a ROS‐positive population, as detected using a flow cytometry analysis.

MitoSOX Red (5 μM, 30 min) [#M36008] (Thermo Fisher Scientific), a mitochondrial superoxide sensitive probe, was used for MitoSOX detection (Shiau et al. 2022). A high level of MitoSOX‐stained cells was considered indicative of a MitoSOX‐positive population, as detected using a flow cytometry analysis.

### 2.8 Mitochondrial Membrane Potential (MMP) Assay

MMP was detected using the mitochondrial membrane‐specific JC‐1 dye [#420200‐5MGCN] (Sigma‐Aldrich) according to the manufacturer's instructions. It exhibits monomers and polymers for damaged and intact mitochondrial membranes, producing green and red fluorescence. These non‐fixed cells were analyzed using a flow cytometer. Finally, the ratio of green fluorescence proportional to MMP was calculated. This revealed that MMP was depleted when the ratio decreased.

### 2.9 γH2AX and 8‐hydroxy‐2′‐deoxyguanosine (8‐OHdG) DNA Damage Assay

The antibody (1:1000) against γH2AX and 8‐OHdG [#SC‐517348 and #SC‐393871] (Santa Cruz Biotechnology) was applied to detect γH2AX and 8‐OHdG DNA damage using a flow cytometry analysis (Shiau et al. 2022). High levels of γH2AX and 8‐OHdG detected cells were considered indicative of γH2AX‐ and 8‐OHdG‐positive populations, as detected using a flow cytometry analysis.

### 2.10 MAPK Signaling

MAPK (JNK, p38, and ERK) and phospho‐MAPK family antibody sampler kits (#9926 and #9910, Cell Signaling Technology) were used in Western blotting under a 1:1000 dilution.

### 2.11 Statistical Analysis

For all experiments, JMP12 software (SAS Institute) was used to determine the significance between multiple comparisons based on a one‐way analysis of variance (ANOVA) using the Tukey HSD post hoc test. Data are shown as mean ± SD (*n* = 3 for cells and *n* = 6 for animals). Data top‐labeled with non‐overlapping small letters show significance.

## 3 Results

### 3.1 CPC Has Antiproliferative Effects on TNBC Cells In Vitro and In Vivo

**Figure 1:**
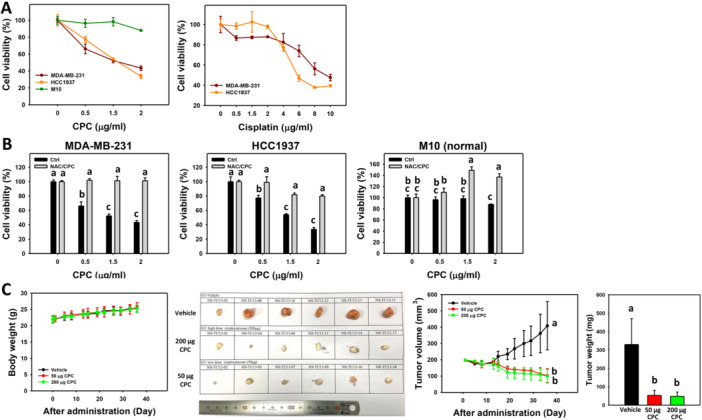
Antiproliferative effects of CPC on TNBC cells in vitro and in vivo. (A) Cell viability of CPC and cisplatin in 24 h ATP assay. Two types of TNBC cells (MDA‐MB‐231 and HCC1937) and normal breast cells (M10) were included. Cells (left) were treated with 0 (vehicle control containing 0.1% DMSO), 0.5, 1.5, and 2 μg/mL of CPC for 24 h. Moreover, cells (right) were treated with 0, 0.5, 1.5, 2, 4, 6, 8, and 10 μg/mL of cisplatin for 24 h. (B) Recovery of CPC‐induced antiproliferation by NAC. Cells were pre‐incubated without or with NAC (10 mM for 1 h) and post‐incubated with CPC for 24 h (2 μg/mL), i.e., CPC and NAC/CPC. Columns labeled without overlapping letters in the same cell line differ significantly (*p *< 0.05) (Tukey HSD post hoc test). Taking panel (B) as an example (MDA‐MB‐231 cells), the “a” (with overlapping letters) labeling the control, NAC, and NAC/CPC (0.5, 1.5, and 2) treatments indicates that their differences were non‐significant. In contrast, the “a, b, and c” (without overlapping letters) labeling the control, CPC 0.5, and CPC 1.5 μg/mL indicates significant differences in multi‐comparisons. Cell experimental data are displayed as means ± SD (*n* = 3). (C) TNBC xenograft tumor suppression of CPC. MDA‐MB‐231‐xenografted mice were treated with CPC (0, 50, and 200 μg: CPC0, CPC50, and CPC200) for 5 weeks. Body weight and tumor volume were monitored twice weekly, and tumor weights were measured at 5 weeks. Animal experiment data are expressed as means ± SD (*n* = 6).

TNBC cells can be differentiated into triple‐negative A (TNA) and triple‐negative B (TNB) types (Dai et al. 2017). TNA cells, such as HCC1937, are enriched with basal markers. TNB cells such as MDA‐MB‐231 are claudin‐low with overexpressed tumor‐invasive and ‐aggressive characteristics. These TNBC cell lines were chosen to evaluate the antiproliferative effect of CPC. In a 24‐h ATP assay, CPC dose‐dependently reduced the viability (%) of the TNBC cells (Figure 1A). In comparison, normal breast cells (M10) showed low cytotoxicity to CPC. Notably, the clinical drug cisplatin inhibited breast cancer cell proliferation to a lesser degree than CPC.

The antiproliferative effects of CPC were alleviated by pretreatment with *N*‐acetylcysteine (NAC), an inhibitor of reactive oxygen species (ROS) (Figure 1B). Accordingly, oxidative stress and apoptosis were involved in the CPC‐induced antiproliferation of the TNBC cells.

Regarding the in vivo response, the antitumor effects of CPC were evaluated in MDA‐MB‐231 tumor‐bearing mice by measuring tumor growth (Figure 1C). Human TNBC (MDA‐MB‐231) cell tumor‐bearing mice were treated with PBS (CPC0), 200 μg CPC (CPC200), and 50 μg CPC (CPC50). CPC was administered once to accompany cells subcutaneously injected into the mice on Day 0. No statistically significant difference was observed in the mean body weight of all treatment groups (Figure 1C). No gross pathological abnormalities were observed during necropsy.

The tumor volumes were recorded on Days 1, 5, 8, 13, 15, 19, 22, 26, 29, 33, and 36 after tumor cell injection (Figure 1C). The results showed that CPC (CPC200 and CPC50) significantly reduced the tumor volume compared with the PBS treatment (CPC0) from Day 15 to Day 36. Moreover, the morphology of the tumors was photographed after they were resected (Figure 1C). There were significant differences in tumor weight between the CPC groups and the PBS group on Day 36. However, there were no differences between the high‐ and low‐dose CPC groups in terms of tumor volume and weight. This study found that the administration of CPC decreased the progression of tumors in an TNBC (MDA‐MB‐231) NOD/SCID model. These results suggest that CPC may have therapeutic potential in the treatment of TNBC.

### 3.2 CPC Disturbs Cell Cycle Progression of TNBC Cells

**Figure 2:**
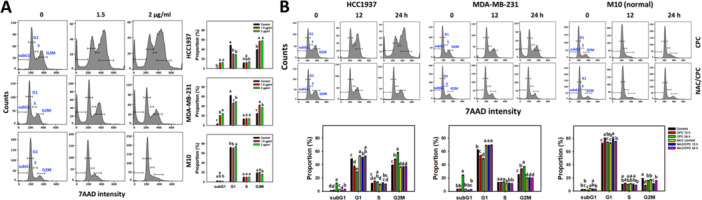
CPC treatment disturbed the cell cycle of TNBC cells. M10 is a normal cell, and others are TNBC cells. (A) Cell cycle detection and statistical analysis of different concentrations of CPC treatment. Cells were treated with 0 (control containing 0.1% DMSO), 1.5, and 2 μg/mL of CPC for 24 h. (B) Cell cycle detection and statistical analysis showed NAC's recovery of different incubation times of CPC treatment. Cells were pre‐incubated without or with NAC (10 mM for 1 h) and post‐incubated with CPC for 12 and 24 h, i.e., CPC and NAC/CPC. For CPC post‐incubation, cells were treated with 0 (control containing 0.1% DMSO), 1.5, and 2 μg/mL of CPC for 24 h. Cell cycle phases such as subG1, G1, S, and G2/M were defined as indicated in the flow cytometry panel. Columns labeled without overlapping letters differ significantly in the same phase of the same cell line (*p* < 0.05) (Tukey HSD post hoc test). Data are expressed as means ± SD (*n* = 3).

Cell cycle histograms of the CPC‐treated TNBC (HCC1937 and MDA‐MB‐231) and normal (M10) cells are displayed (Figure 2A,B). CPC induced greater subG1 accumulation (apoptosis‐like phenomena) in the dose‐ and time‐dependent manners than the control in the TNBC cells (HCC1937 and MDA‐MB‐231). Moreover, the pattern shows that CPC disturbed the cell cycle progression such as the G1 decrease and G2/M arrest of the TNBC cells, partially recovering the cell distribution similarly to the control with NAC pretreatment (NAC/CPC) (Figure 2B). In comparison, the normal cells (M10) showed little changes in the cell cycle and low subG1 accumulation (Figure 2A,B).

### 3.3 CPC Triggers Apoptosis of TNBC Cells

**Figure 3:**
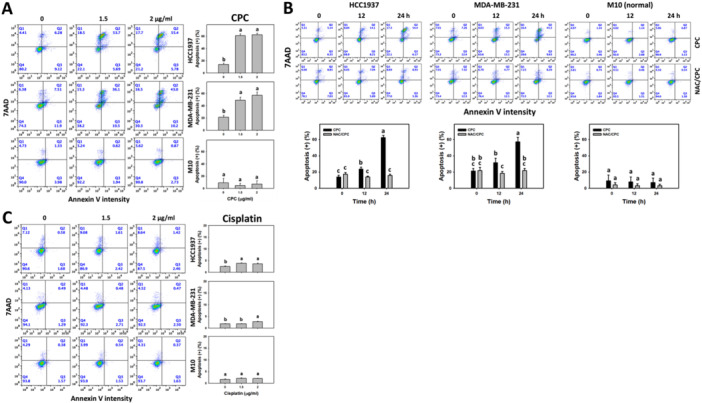
CPC induces apoptosis (annexin V) in TNBC cells. M10 is a normal cell, and others are TNBC cells. (A) Apoptosis (annexin V) detection and statistical analysis of different concentrations of CPC treatment. Cells were treated with 0 (control containing 0.1% DMSO), 1.5, and 2 μg/mL of CPC for 24 h. Annexin V‐positive population indicated apoptosis (+). (B) Apoptosis (annexin V) detection and statistical analysis showed NAC's recovery of different incubation times of CPC‐induced apoptosis. Cells were pre‐incubated without or with NAC (10 mM for 1 h) and post‐incubated with CPC for 0, 12, and 24 h (2 μg/mL), i.e., CPC and NAC/CPC. (C) Apoptosis (annexin V) detection and statistical analysis of different concentrations of cisplatin treatment. Cells were treated with 0, 1.5, and 2 μg/mL of cisplatin for 24 h. Columns labeled without overlapping letters differ significantly in the same cell line (*p* < 0.05) (Tukey HSD post hoc test). Data are expressed as means ± SD (*n* = 3).

To further confirm the CPC‐induced apoptosis for the subG1 accumulation (apoptosis‐like phenomena) in the TNBC cells, an annexin V/7AAD analysis was performed. Annexin V/7AAD profiles of the CPC‐treated TNBC cells (HCC1937 and MDA‐MB‐231) were obtained for different CPC concentrations and incubation times (Figure 3A,B). Various concentrations of CPC induced more apoptosis (+) (%) (annexin V (+)/7AAD (+/−)) than the control in the TNBC cells (Figure 3A). In addition, CPC induced more apoptosis (+) (%) in the TNBC cells over time, which was partially suppressed by NAC pretreatment (Figure 3B). Notably, the clinical drug cisplatin induced breast cancer cell apoptosis to a lesser degree than CPC at the same concentrations (0, 1.5 and 2 μg/mL) (Figure 3C). In comparison, the normal cells (M10) showed little changes in annexin V for CPC and cisplatin treatments (Figure 3B,C).

### 3.4 CPC Activates Apoptotic Signaling in TNBC Cells (Western Blotting)

**Figure 4:**
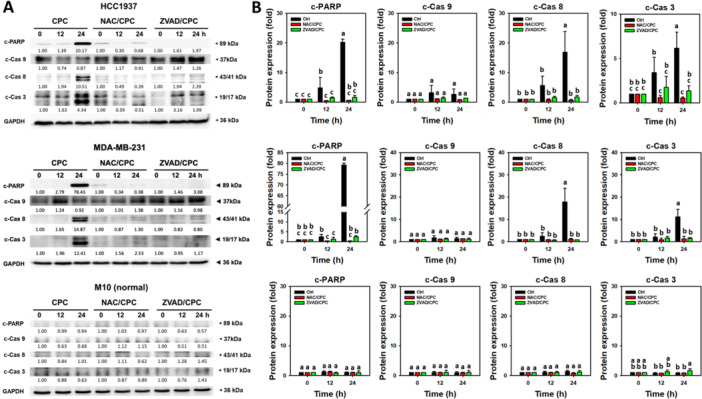
CPC induces extrinsic apoptosis (PARP, caspase 3, and caspase 8 activation) but not intrinsic apoptosis (caspase 9) in TNBC cells. With pretreatments of vehicle, NAC (10 mM for 1 h), or Z‐VAD (50 μM for 2 h), cells were treated with 2 μg/mL of CPC for 0, 12, and 24 h. M10 is a normal cell, and others are TNBC cells. (A) Expression of cleaved forms of PARP, caspase 3, and caspase 8 were detected using Western blotting. (B) Statistical analysis. Columns labeled without overlapping letters differ significantly in the same cell line (*p* < 0.05) (Tukey HSD post hoc test). Data are expressed as means ± SD (*n* = 3). c‐PARP, c‐Cas 3, c‐Cas 8, and c‐Cas 9 indicate the cleaved forms of PARP, caspases 3, 8, and 9.

Apoptotic signaling activation, such as the activation of PARP and caspases 3, 8, and 9, provides significant steps for the induction of apoptosis (Boice and Bouchier‐Hayes 2020). To further confirm CPC‐induced apoptosis (annexin V) in the TNBC cells (HCC1937 and MDA‐MB‐231) (Figure 3), the activation of PARP and caspases 3, 8, and 9 for the induction of apoptosis was examined (Figure 4). CPC induced a greater activation of caspases 3 and 8 than caspase 9 in the TNBC cells after 24 h of treatment, which was suppressed by NAC or ZVAD pretreatment. In comparison, the normal cells (M10) showed little changes in caspases 3, 8, and 9.

### 3.5 CPC Activates Caspases 3 and 8 in TNBC Cells (Flow Cytometry or Luminescence Assays)

**Figure 5:**
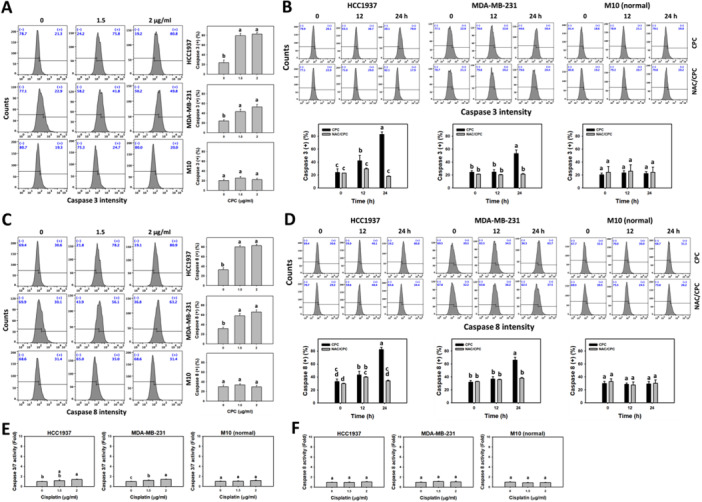
CPC induces apoptosis (caspase 3 and 8 activation) in TNBC cells. M10 is a normal cell, and others are TNBC cells. (A, C) Caspase 3/8 detection and statistical analysis for different concentrations of CPC treatment. Cells were treated with 0 (control containing 0.1% DMSO), 1.5, and 2 μg/mL of CPC for 24 h. Symbol (+) represents caspase 3/8 (+) in flow cytometry. (B, D) Caspase 3/8 detection and statistical analysis showed NAC's recovery of different incubation times of CPC‐induced apoptosis in flow cytometry. Cells were pre‐incubated without or with NAC (10 mM for 1 h) and post‐incubated without or with CPC for 0, 12, and 24 h (2 μg/mL), i.e., CPC and NAC/CPC. (E, F) Caspase 3/7 and 8 detection and statistical analysis for different concentrations of cisplatin treatment in luminescence assays. Cells were treated with 0, 1.5, and 2 μg/mL of cisplatin for 24 h. Columns labeled without overlapping letters differ significantly in the same cell line (*p* < 0.05) (Tukey HSD post hoc test). Data are expressed as means ± SD (*n* = 3).

To further confirm CPC‐induced extrinsic apoptosis in the TNBC cells, the activation of caspases 3 and 8 for the induction of apoptosis was examined in the present study. The different concentrations of CPC induced more active caspases 3 and 8 (+) (%) in the TNBC cells than the control (Figure 5A,C). In addition, CPC induced more active caspases 3 and 8 (+) (%) in the TNBC cells over time, which were partially suppressed by NAC pretreatment (Figure 5B,D). Specifically, CPC induced a greater activation of caspases 3 and 8 than the control in the TNBC cells over time. Notably, the clinical drug cisplatin induced caspase 3/7 and caspase 8 activations of breast cancer cells to a lesser degree than CPC at the same concentrations (0, 1.5 and 2 μg/mL) (Figure 5E,F). In comparison, the normal cells (M10) showed little change in caspases 3/7 and 8 for CPC and cisplatin treatments (Figure 5A,C,E,F).

### 3.6 CPC Overexpresses ROS and MitoSOX in TNBC Cells

**Figure 6:**
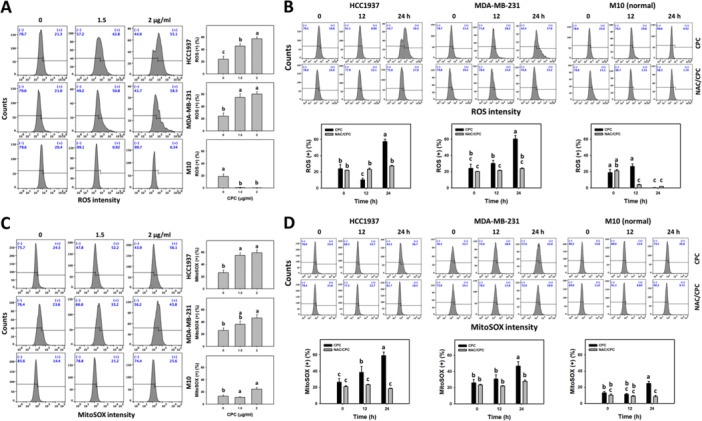
CPC overexpresses ROS and MitoSOX in TNBC cells. M10 is a normal cell, and others are TNBC cells. (A, C) ROS detection and statistical analysis of different concentrations of CPC treatment. Cells were treated with 0 (control containing 0.1% DMSO), 1.5, and 2 μg/mL of CPC for 24 h. Symbol (+) represents high levels of ROS. (B, D) ROS and MitoSOX detection and statistical analysis showed NAC's recovery of different incubation times of CPC‐induced ROS and MitoSOX. Cells were pre‐incubated without or with NAC (10 mM for 1 h) and post‐incubated with CPC for 0, 12, and 24 h (2 μg/mL), i.e., CPC and NAC/CPC. Columns labeled without overlapping letters differ significantly in the same cell line (*p* < 0.05) (Tukey HSD post hoc test). Data are expressed as mean ± SD (*n* = 3).

As demonstrated above, NAC reverts several CPC responses. Therefore, the ROS and MitoSOX profiles of the CPC‐treated TNBC cells (HCC1937 and MDA‐MB‐231) were investigated, and the results are shown in Figure 6A,C. Different concentrations of CPC induced more ROS and MitoSOX (+) (%) than the control in the TNBC cells (Figure 6B,D). In addition, CPC generated more ROS and MitoSOX (+) (%) in the TNBC cells over time, which were partially suppressed by NAC pretreatment. In comparison, the normal cells (M10) showed little changes in ROS and MitoSOX.

### 3.7 CPC Depletes MMP in TNBC Cells

**Figure 7:**
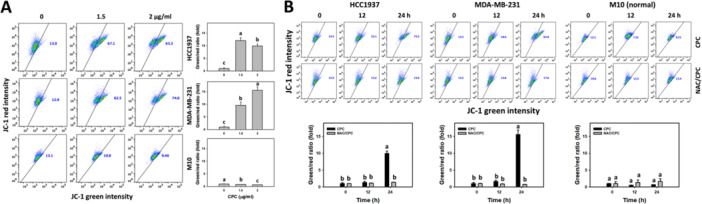
CPC triggers MMP depletion in TNBC cells. M10 is a normal cell, and others are TNBC cells. (A) MMP detection and statistical analysis of different concentrations of CPC treatment. Cells were treated with 0 (control containing 0.1% DMSO), 1.5, and 2 μg/mL of CPC for 24 h. JC‐1 red and green represent the polymers and monomers of JC‐1. High JC‐1 ratio (green/red) indicates MMP depletion. (B) MMP detection and statistical analysis showed NAC's recovery of different incubation times of CPC‐induced MMP depletion. Cells were pre‐incubated without or with NAC (10 mM for 1 h) and post‐incubated with CPC for 0, 12, and 24 h (2 μg/mL), i.e., CPC and NAC/CPC. Columns labeled without overlapping letters differ significantly in the same cell line (*p* < 0.05) (Tukey HSD post hoc test). Data are expressed as means ± SD (*n* = 3).

MMP depletion is associated with mitochondrial disorder and oxidative stress (Maharjan et al. 2014). Therefore, the MMP profiles of the CPC‐treated TNBC cells (HCC1937 and MDA‐MB‐231) were obtained (Figure 7A,B). The different concentrations of CPC induced a higher MMP ratio (green/red), i.e., MMP depletion, in the TNBC cells than the control (Figure 7A). In addition, CPC yielded a higher ratio (green/red) in the TNBC cells over time, which was partially suppressed by NAC pretreatment (Figure 7B). In comparison, the normal cells (M10) showed little changes in MMP.

### 3.8 CPC Causes γH2AX and 8‐OHdG DNA Damage in TNBC Cells

**Figure 8:**
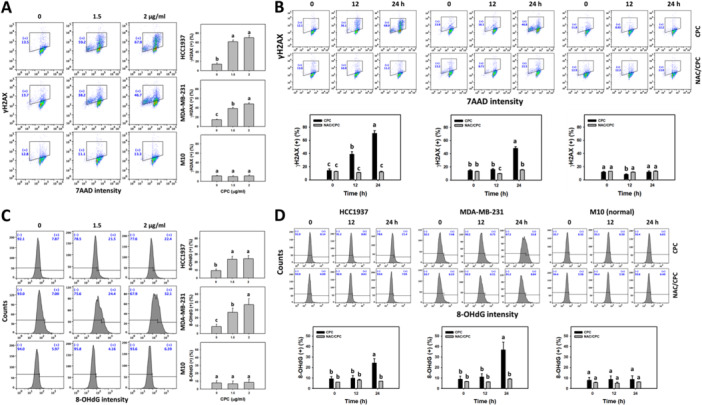
CPC causes γH2AX and 8‐OHdG DNA damage in TNBC cells. M10 is a normal cell, and others are TNBC cells. (A, C) γH2AX and 8‐OHdG DNA damage detection and statistical analysis of different concentrations of CPC treatment. Cells were treated with 0 (control containing 0.1% DMSO), 1.5, and 2 μg/mL of CPC for 24 h. The symbol (+) represents high levels of γH2AX and 8‐OHdG. (B, D) γH2AX and 8‐OHdG detection and statistical analysis showed NAC's recovery of different incubation times of CPC‐induced γH2AX and 8‐OHdG DNA damage. Cells were pre‐incubated without or with NAC (10 mM for 1 h) and post‐incubated with CPC for 0, 12, and 24 h (2 μg/mL), i.e., CPC and NAC/CPC. Columns labeled without overlapping letters differ significantly in the same cell line (*p* < 0.05) (Tukey HSD post hoc test). Data are expressed as means ± SD (*n* = 3).

γH2AX and 8‐OHdG are DNA double‐strand breaks (Mah et al. 2010) and oxidative DNA damage markers (Kumar et al. 2012). The γH2AX and 8‐OHdG profiles of the CPC‐treated TNBC cells (HCC1937 and MDA‐MB‐231) were obtained (Figure 8A,C). The different concentrations of CPC induced more γH2AX and 8‐OHdG (+) (%) than the control in the TNBC cells (Figure 8A,C). In addition, CPC yielded more γH2AX and 8‐OHdG (+) (%) in the TNBC cells over time, which was partially suppressed by NAC pretreatment (Figure 8B,D). In comparison, normal cells (M10) showed little changes in γH2AX and 8‐OHdG.

### 3.9 The Role of JNK and p38 on CPC‐Caused Antiproliferation, Apoptosis, and Caspase 3/8 Activation

**Figure 9:**
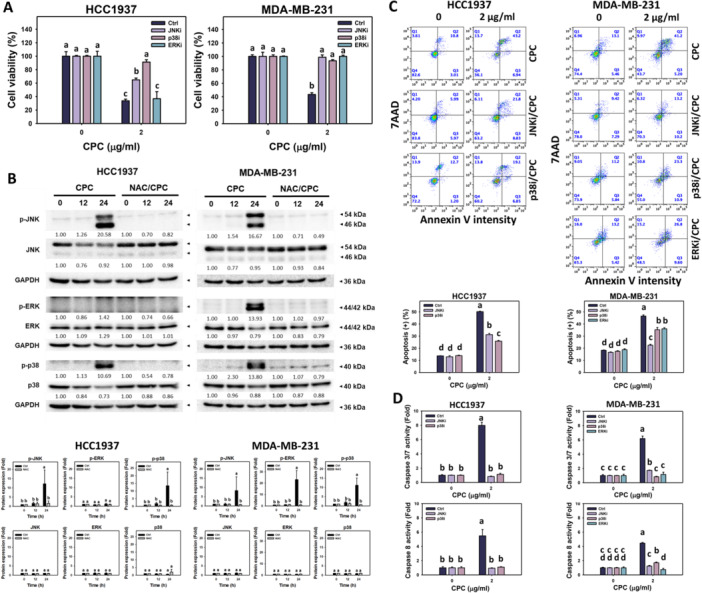
CPC causes MAPK activation in TNBC cells. Cells were pre‐incubated without or with NAC (10 mM for 1 h) or MAPK inhibitors (JNK, p38 or ERK) and post‐incubated with CPC for 0, 12, and 24 h (2 μg/mL). (A) Cell viability of MAPK inhibitors in CPC‐treated TNBC cells. (B) Phosphorylation of MAPKs by CPC on TNBC cells. (C) Apoptosis of JNKi/CPC, p38i/CPC, and ERKi/CPC in TNBC cells. (D) Caspase 3/7 and 8 activations of JNKi/CPC, p38i/CPC, and ERKi/CPC in TNBC cells. Columns labeled without overlapping letters differ significantly in the same cell line (*p* < 0.05) (Tukey HSD post hoc test). Data are expressed as means ± SD (*n* = 3).

Three members of the MAPK family (JNK, ERK, and p38), are generally modulated by ROS (Jeong et al. 2014; Runchel et al. 2011). The contribution of the MAPKs to CPC‐induced antiproliferative effects was explored. The CPC‐suppressed proliferation of TNBC cells (HCC1937 and MDA‐MB‐231) was attenuated by JNKi (SP600125) and p38i (SB203580). While the ERK inhibitor (ERKi) (PD98059) attenuated CPC‐induced antiproliferation on MDA‐MB‐231 cells but showed a minor change on HCC1937 cells (Figure 9A).

Moreover, the MAPK signaling in CPC‐treated TNBC cells was assessed. CPC upregulated higher phosphorylated JNK and p38 (p‐JNK and p‐p38) expressions in TNBC cells (HCC1937 and MDA‐MB‐231) than in control, suppressed by NAC (Figure 9B). This indicates that CPC promotes the phosphorylation of JNK and p38 in these two kinds of TNBC cells through the generation of ROS. While p‐ERK was upregulated by CPC and attenuated by NAC in MDA‐MB‐231 cells, p‐ERK did not change by CPC in HCC1937 cells, compared to ERK levels.

Next, the impact of apoptosis and caspase signaling needed to be assessed by JNKi and p38i (HCC1937) and JNKi, p38i, and ERKi (MDA‐MB‐231). For HCC1937 cells, JNKi and p38i attenuated CPC‐induced apoptosis (annexin V) (Figure 9C) and caspase 3/7 and caspase 8 activations (Figure 9D). In comparison, JNKi, p38i, and ERKi attenuated CPC‐induced apoptosis (annexin V) (Figure 9C), caspase 3/7, and caspase 8 activations (Figure 9D) in MDA‐MB‐231 cells.

## 4 Discussion

Anticancer reports related to CPC (Chen et al. 2010; Kurniadewi et al. 2010; Ren et al. 2014) are rare. Antiproliferation has been found in some cancers (leukemia, prostate, and colon cancers) but not in breast cancer. Moreover, most of these studies aimed to identify new compounds and provided only cytotoxicity information for the anticancer function. The detailed anticancer mechanisms in CPC and its cytotoxicity to normal cells have rarely been investigated. The present study confirms that CPC has antiproliferative effects on different types of TNBC cells, such as TNA (HCC1937) and TNB (MDA‐MB‐231), and it explores the precise mechanisms of its anti‐TNBC action.

### 4.1 CPC Shows Antiproliferation Effects on TNBC Cells Depending on Oxidative Stress

In a previous study, CPC exhibited selective antiproliferation effects on oral cancer cells without cytotoxicity to normal oral cells, relying on oxidative stress (Chang et al. 2016). Additionally, in a 24 h MTS assay, CPC showed IC_50_ values of 11.63 and 3.91 μg/mL in oral cancer cells (Ca9‐22 and CAL 27) (Chang et al. 2016). In 48 h SRB assay, CPC showed IC_50_ values of 0.65 and 0.96 μg/mL in androgen‐independent prostate cancer cells (LNCaP and DU‐145) (Chen et al. 2010) and 0.39 and 0.45 μg/mL in liver cancer cells (SK‐Hep‐1 and HuH‐7) (Yu et al. 2023). However, these studies did not evaluate the effects of in vivo anticancer.

In the present study, in a 24 h ATP assay, CPC showed IC_50_ values of 1.60 and 1.63 μg/mL in TNBC cell lines (HCC1937 and MDA‐MB‐231) (Figure 1A). These results suggest that CPC had antiproliferative effects on both TNA and TNB types but had little cytotoxicity to normal cells (M10). Regarding the in vivo evaluation, CPC exhibited tumor growth‐suppressive effects and tumor regression in the mice subcutaneously inoculated with TNBC (MDA‐MB‐231 cells) without affecting body weight (Figure 1C).

Cisplatin is a first‐line drug for breast cancer, especially for TNBC. However, cisplatin therapy is hampered by its side effects (Romani 2022). Cisplatin showed IC_50_ values of 100.5 and 20.7 μg/mL in a 48‐h ATP assay in HCC1937 cells (Deng et al. 2014) and MDA‐MB‐231 (Shiau et al. 2025), respectively. Moreover, cisplatin showed IC_50_ values of 5.8 and 9.4 μg/mL in a 24‐h ATP assay in HCC1937 and MDA‐MB‐231 cells (Figure 1A)., i.e., 3.6 and 5.8 times more sensitive for HCC1937 and MDA‐MB‐231 cells, respectively, based on the ratio of their IC_50_ values (cisplatin/CPC = 5.8/1.60 and 9.4/1.63) using a 24‐h ATP assay. Consistently, cisplatin was less effective at inducing apoptosis (Figure 3C) and activating caspases 3/7 and 8 (Figure 5E,F) than CPC at the same concentrations (0, 1.5 and 2 μg/mL). Thus, CPC may exhibit more effective than cisplatin in the current study.

Moreover, NAC pretreatment rescued CPC‐induced antiproliferation in TNBC, indicating that CPC induces oxidative stress to inhibit the proliferation of TNBC cells. In comparison, the CPC effects on prostate (Chen et al. 2010) and liver (Yu et al. 2023) cancer cells did not assess the change and impact of ROS.

### 4.2 CPC Overexpresses Oxidative Stress in TNBC Cells

As NAC reverts CPC‐induced antiproliferation, oxidative stress plays a vital role in TNBC antiproliferation. CPC overexpressed both ROS and MitoSOX. Moreover, MMP depolarization is associated with oxidative stress generation (Maharjan et al. 2014). CPC also suppressed MMP by inducing MMP depolarization (MMP depletion) in the TNBC cells. Thus, CPC induces oxidative stress responses in TNBC cells. NAC pretreatment further confirmed these oxidative stress inductions; i.e., NAC reverts the changes in CPC‐induced oxidative stresses in TNBC cells.

### 4.3 CPC‐Induced Apoptosis and DNA Damage in TNBC Cells Depend on Oxidative Stress

Oxidative stress is known to activate apoptosis (Redza‐Dutordoir and Averill‐Bates 2016). In the present study, CPC caused subG1 cell cycle accumulation, indicating apoptosis‐like changes (Semaan et al. 2016) in the TNBC cells. Moreover, CPC promoted annexin V‐detected apoptosis and activated caspases 3 and 8 rather than caspase 9. Thus, CPC induces extrinsic apoptotic responses in TNBC cells. Furthermore, NAC reverted the changes in CPC‐induced apoptosis and its associated signaling in the TNBC cells. These results indicate that CPC triggers apoptosis through oxidative stress in TNBC cells. CPC also activates PARP and caspase 3 in liver cancer cells (Yu et al. 2023), suggesting that CPC has apoptosis‐inducing potential for several types of cancer cells.

Moreover, ROS overexpression in cells leads to oxidative stress (Salehi et al. 2018), which causes DNA damage (Srinivas et al. 2019). Oxidative stress‐modulating agents also induce oxidative DNA damage (Salehi et al. 2018). In the present study, CPC promoted oxidative DNA damage in the TNBC cells by detecting γH2AX and 8‐OHdG. Thus, CPC induces DNA damage responses in TNBC cells. Moreover, NAC reverted the changes in CPC‐induced DNA damage. This indicates that CPC triggers DNA damage through oxidative stress in TNBC cells.

### 4.4 The Role of MAPK in Modulating Antiproliferation, ROS, Apoptosis, and Caspase Activation

Proliferation and apoptosis are modulated by the interaction between ROS and MAPK signaling. Regarding JNK and p38 activation, their antiproliferative role acting on cancer cells has been reported. For example, furanodienone (Jiang et al. 2017) and berberine (Hsu et al. 2007) inhibits colon cancer cell proliferation by upregulating p‐JNK and p‐p38, attenuated by NAC. Similarly, CPC‐induced antiproliferation of TNBC cells (HCC1937 and MDA‐MB‐231) was attenuated by the inhibition of JNK and p38 (Figure 9A). CPC upregulates p‐JNK and p‐p38 in these TNBC cells, which were suppressed by NAC. Accordingly, the contributions of JNK and p38 in the CPC‐triggered apoptosis of these TNBC cells were evaluated. CPC‐triggered annexin V‐detected apoptosis was suppressed by JNKi and p38i in these TNBC cells (Figure 9C). Furthermore, JNK and p38 promote caspase 3 activation triggered by gemcitabine (Teraishi et al. 2005) and H_2_O_2_ (Pearl‐Yafe et al. 2004) in lung cancer and Fanconi anemia C cells, respectively. Similarly, JNKi and p38i suppressed caspase 3/8 activations by CPC in these TNBC cells (HCC1937 and MDA‐MB‐231) (Figure 9D).

Notably, different kinds of TNBC cells may exhibit differential regulation of certain MAPK members, such as ERK. ERK may promote proliferation or apoptosis, exhibiting a dual role for antiproliferation of cancer cells (Sugiura et al. 2021). For instance, ERK overexpression enhances tumorigenesis, which is attenuated by ERK inhibition (Guo et al. 2020), thereby demonstrating the pro‐survival function of ERK. Similarly, furanodienone inhibits colon cancer cell proliferation by downregulating p‐ERK, attenuated by NAC (Jiang et al. 2017). Regarding HCC1937 cells, the p‐ERK was not induced by CPC (Figure 9B), and CPC‐induced antiproliferation was not attenuated by ERKi (Figure 9A).

In contrast, ERK may exhibit pro‐apoptotic function. For example, triptolide inhibits TNBC (MDA‐MB‐231) cell proliferation by upregulating ERK and caspase 3 activations (Tan and Chiu 2013). Regarding MDA‐MB‐231 cells, the p‐ERK was induced by CPC (Figure 9B). Additionally, CPC‐induced antiproliferation (Figure 9A), annexin V (Figure 9C), and caspases 3/8 (Figure 9D) of MDA‐MB‐231 cells were attenuated by ERKi. This suggests that ERK plays a pro‐apoptotic role in CPC‐treated MDA‐MB‐231 cells.

These results indicate that CPC differentially induces MAPK‐mediated apoptosis and caspase 3/8 activation in TNBC cells (HCC1937 and MDA‐MB‐231). Therefore, CPC drives apoptosis mediated by the ROS‐MAPK axis, resulting in the antiproliferative effects observed in TNBC cells.

## 5 Conclusions

**Figure 10:**
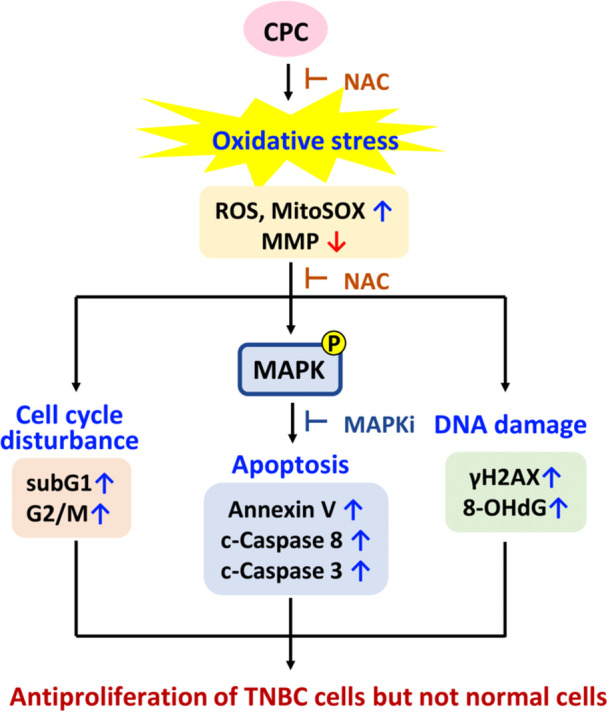
Overview of CPC's anti‐TNBC cell proliferation effect and mechanism. CPC caused selective oxidative stress, MAPK activation, apoptosis, and DNA damage in TNBC cells, leading to the selective inhibition of TNBC cell proliferation compared with normal cells. These selective anti‐TNBC effects of CPC were dependent on oxidative stress, confirmed by the pretreatments of NAC or MAPK inhibitors.

TNBC does not contain the common targets (ER, PR, and HER2) and has a weaker response to traditional targeted therapies than non‐TNBC. The present investigation confirmed that *C. concinna*‐derived CPC exhibits antiproliferation effects on several types of TNBC cells depending on oxidative stress. CPC also showed suppressing effects on tumor growth in a TNBC‐xenografted mouse model. CPC generated both cellular and mitochondrial stresses. Moreover, CPC effectively induced extrinsic apoptosis by activating caspase 8 and 3 signaling and caused oxidative DNA damage in TNBC cells (HCC1937 and MDA‐MB‐231) (Figure 10). CPC also promotes more ROS‐mediated JNK and p38 phosphorylation in TNBC cells, confirmed by NAC. This JNK and p38 phosphorylation is important in enhancing antiproliferation, apoptosis, and caspase 3/8 activation in these TNBC cells, as confirmed by JNKi and p38i. While ERK exhibits differential regulation by CPC in different TNBC cells (HCC1937 and MDA‐MB‐231). Taken together, we demonstrate here for the first time that CPC causes the antiproliferation of several types of TNBC cells through oxidative stress‐dependent apoptosis and DNA damage involving MAPK modulation.

## Author Contributions

Conceptualization: Ya‐Ting Chuang, Ammad Ahmad Farooqi, Jun‐Ping Shiau, and Hsueh‐Wei Chang. Data curation: Ya‐Ting Chuang. Formal analysis: Ya‐Ting Chuang. Methodology: Wangta Liu, Tsu‐Ming Chien, and Hsun‐Shuo Chang. Supervision: Jun‐Ping Shiau and Hsueh‐Wei Chang. Writing – original draft: Ya‐Ting Chuang, Jun‐Ping Shiau, and Hsueh‐Wei Chang. Writing – review and editing: Ya‐Ting Chuang, Jun‐Ping Shiau, and Hsueh‐Wei Chang.

## Conflicts of Interest

The authors declare no conflicts of interest.

## Data Availability

The data that support the findings of this study are available from the corresponding author upon reasonable request.
